# Multiple exposure sources and occupational fatigue profiles among healthcare workers: a cross-sectional latent profile analysis

**DOI:** 10.3389/fpubh.2026.1811642

**Published:** 2026-05-20

**Authors:** Lanhui Tan, Ruyi Zhang, Yifan Liao, Shi Liu, Fei Fang, Li Liu, Bilong Feng, Ying Wang

**Affiliations:** 1 Department of Nursing, Zhongnan Hospital of Wuhan University, Wuhan, Hubei, China; 2 Department of Infectious Diseases, Zhongnan Hospital of Wuhan University, Hubei, China; 3 Department of Building Science, Tsinghua University, Beijing, China; 4 Jingmen Central Hospital, Jingzhou, Hubei, China; 5 Hubei Engineering Center for Infectious Disease Prevention, Control and Treatment, Wuhan, Hubei, China; 6 Department of Infection Prevention and Control Management, Zhongnan Hospital of Wuhan University, Wuhan, Hubei, China

**Keywords:** exposure sources, healthcare worker, latent profile analysis, occupational fatigue, risk factors

## Abstract

**Background:**

Occupational fatigue is a complex and widespread issue among healthcare workers, yet its heterogeneous manifestations remain inadequately studied. This study aimed to identify distinct fatigue profiles and examine the multifaceted determinants that differentiate these profiles.

**Methods:**

A cross-sectional survey was conducted among 734 healthcare workers. The assessment was conducted using the newly developed Healthcare Worker Occupational Fatigue Scale that has undergone reliability and validity tests. Latent profile analysis was used to identify occupational fatigue subgroups, and multiple logistic regression analysis was conducted to explore the influencing factors of each subgroup.

**Results:**

Latent profile analysis identified four distinguishable occupational fatigue subgroups: the compensated group (17.97%), the prodromal-symptomatic group (35.29%), the decompensated diffuse group (37.87%), and the systemic crisis group (8.86%). Multivariate logistic regression analysis revealed that perceived workload, department affiliation, number of night shifts per month, low intention to stay, noisy environment, commuting time, and individual-level factors, including gender and health status, were significant risk factors for occupational fatigue.

**Conclusion:**

Occupational fatigue among healthcare workers exhibits substantial heterogeneity and can be categorized into four distinct profiles, with multiple contributing factors. It is necessary to adopt hierarchical and personalized intervention strategies based on precise subgroup characteristics, such as systematically reducing the workload and optimizing the acoustic environment for the severe fatigue group, in order to effectively alleviate the occupational fatigue of healthcare workers.

## 1 Introduction

Occupational fatigue is the exhaustion that arises during, after, or in association with work (1). High demands, heavy workloads, staffing shortages, shift work, and emotional labor pressures make healthcare workers (HCWs) a high-risk group (2, 3). Related studies have reported prevalence rates ranging from 21.6 to 91.9%, with variations stemming from assessment tools, sample size, and social environments (4). Accumulating evidence indicates that occupational fatigue significantly affects an individual’s health-related quality of life. In extreme cases, it may result in fatality, a phenomenon known as “Karoshi” in Japan (5), which translates to “overwork death” or “death from overwork.” Furthermore, fatigued HCWs may exhibit reduced concentration, delayed reaction times, and impaired decision-making abilities, thereby threatening patient safety and work efficiency (6–8).

Numerous studies have consistently indicated that occupational fatigue among HCWs is not caused by a single stressor but rather results from the combined and synergistic effects of multiple occupational exposure factors. The Systems Engineering Initiative for Patient Safety (SEIPS) model (1) indicates that occupational fatigue stems from the interaction between the individual and the entire work system (i.e., person, organization, technology and tools, tasks, and environment). Focusing on the characteristics of HCWs’ professional tasks, key exposure sources include load exposure and situational exposure. Load exposure, characterized by prolonged high-intensity work tasks and extended shift schedules, has been associated with a 25% increase in instantaneous fatigue levels among staff (9). Situational exposure refers to acute stressors that HCWs frequently encounter during their duties, such as emergency rescues and death-related incidents. During the 2019 pandemic, emotional exhaustion rates increased by 27%, severely compromising mental health (10). Notably, environmental exposure has also emerged as a significant yet overlooked determinant. According to the SEIPS model, the physical environment (particularly lighting, noise and spatial layout) directly influences the circadian rhythm, cognitive load and emotional state of workers, thereby functioning as an active contributor to fatigue (1). Empirical evidence corroborates this relationship. An interventional study by Cyr et al. (11) demonstrated that evening light exposure interventions effectively delayed circadian rhythms among night-shift workers, reducing fatigue-related error rates by 67%.

In summary, many studies have focused on the potential risks of occupational fatigue among HCWs. A key methodological limitation of prior research lies in the fatigue assessment tools employed. Previous studies have assessed occupational fatigue using well-established instruments, including the Multidimensional Fatigue Inventory (MFI), Three-Dimensional Work Fatigue Inventory (3D-WFI), and Fatigue Scale-14 (FS-14) (12). Although these tools offer valuable approaches to fatigue measurement, their applicability to HCWs remains limited. Specifically, prior studies have not sufficiently considered the unique nature of work tasks and environmental scenarios that contribute to occupational fatigue among HCWs. Owing to the inherent vulnerability of the patients, the continuous nature of work schedules, and sustained high-intensity occupational demands, HCWs face an elevated risk of developing occupational fatigue. These multidimensional occupational exposures cannot be precisely captured by generic scales, thereby resulting in a disconnect between the current understanding of fatigue among HCWs and the complex realities of their actual work settings. Importantly, studies relying on generic instruments have commonly reported mean-level fatigue trends or categorized participants into ordinal severity groups (e.g., mild, moderate, and severe fatigue) (13). This may overlook the multi-dimensional heterogeneity of HCWs’ occupational fatigue. Consequently, the detailed profile of occupational fatigue remains difficult to elucidate, which in turn hinders the sustainable implementation and dissemination of interventions.

Consequently, a recent study systematically developed and psychometrically validated the Healthcare Worker Occupational Fatigue Scale (HWOFS) grounded in the conceptual framework of occupational fatigue (14). The HWOFS comprises five dimensions: physical, cognitive and emotional, social, sleep-related, and auditory fatigue. It covers and integrates the occupational contexts of HCWs and provides a standardized and professional metric for assessing occupational fatigue among HCWs. The present study aimed to use the HWOFS in a cross-sectional survey spanning 34 departments. Latent profile analysis (LPA) was employed to delineate fatigue profiles across different dimensions of HCWs, which can accurately capture both quantitative differences and multidimensional qualitative distinctions between individuals. This analytical approach facilitates the exploration of subgroup characteristics and heterogeneity, thereby enabling precise identification of high-risk populations. The study further examined the current state of occupational fatigue and its influencing factors, aiming to inform scientific management of occupational load among HCWs, enhance healthcare system resilience, and promote sustainable human resource development.

## 2 Methods

This study received ethical approval from the Medical Ethics Committee of Zhongnan Hospital of Wuhan University (Number: 2025126 K) and complied with the Declaration of Helsinki. All participants provided informed consent prior to participation.

### 2.1 Participants

From June 10th to 25th, 2025, participants were recruited from a tertiary Grade A general hospital in Hubei Province, China. The inclusion criteria were: (1) doctors, nurses, pharmacists, laboratory or examination personnel, technicians, administrative staff, and manual workers; (2) active employment during the investigation period; and (3) provision of informed consent and voluntary participation. The exclusion criteria were: (1) individuals who are temporarily away from this hospital due to off-site training or further education elsewhere; (2) those who did not complete the full questionnaire; (3) individuals who experienced significant life changes in the past month, such as bereavement, severe physical illness (e.g., recent surgery, postural or musculoskeletal disorders, neurological disorders); and (4) participants currently receiving psychological treatment or counseling.

### 2.2 Sample size

For this descriptive cross-sectional study, the sample size was calculated using the formula (15):


$$N={\left(\frac{U_{\alpha }S}{\delta }\right)}^{2}$$

**Figure 1:**
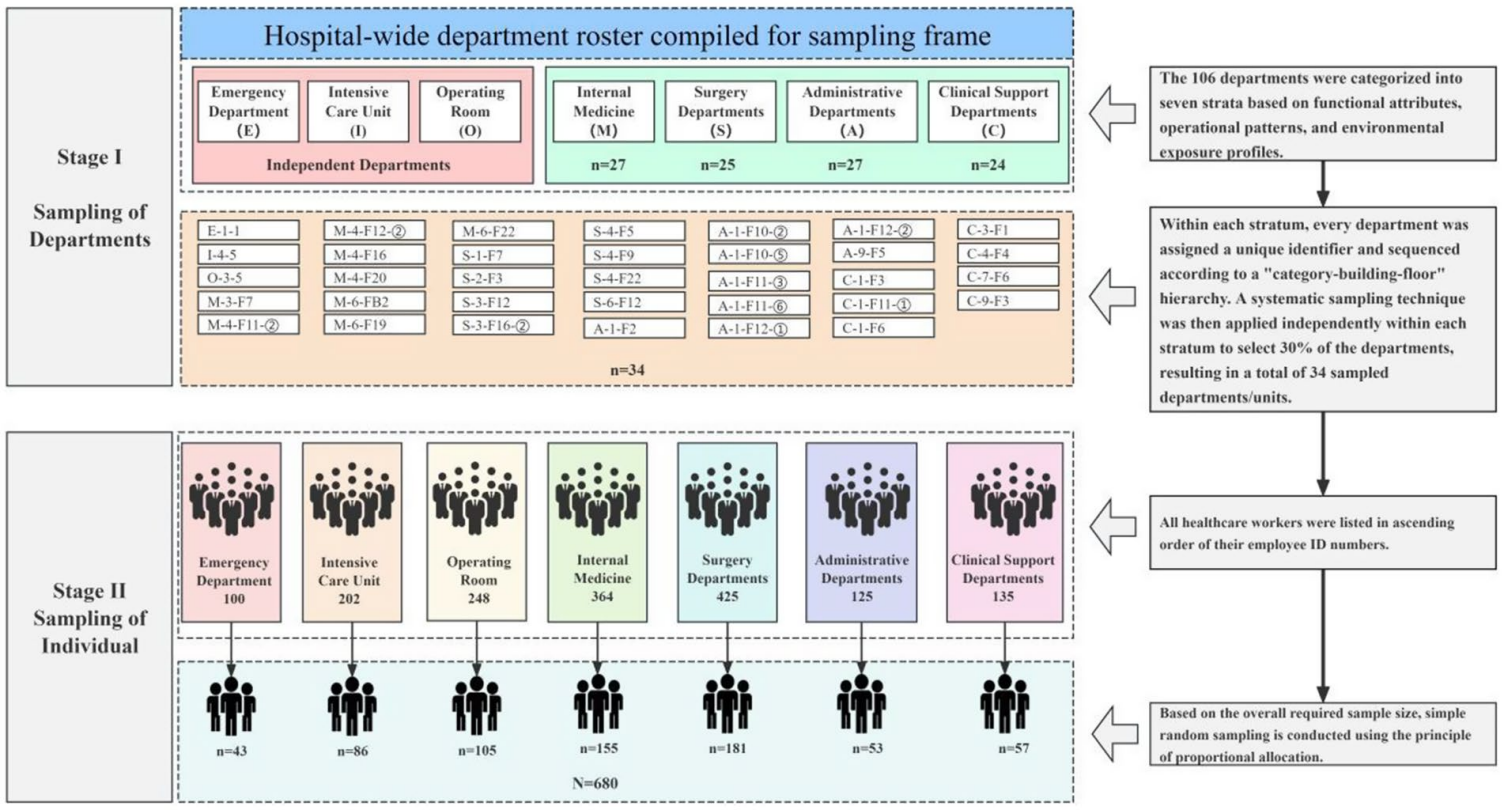
Sampling procedure.

At a 95% confidence interval, $U_{\alpha }$ =1.96, δ represents the absolute error or precision (3 in this study), and S is the standard deviation of occupational fatigue among HCWs from a pilot study (25.202) (14). According to this formula, the theoretical sample size was 272. To ensure representativeness in alignment with the hospital staff structure, a multistage, stratified random sampling method was employed. The specific sampling procedure is illustrated in Figure 1. Considering a design effect (DEFF) of 2 and a 20% anticipated invalid response rate (16), the final required sample size was at least 680.

### 2.3 Measurements

#### 2.3.1 General demographic questionnaire

Demographic information (i.e., age, gender, educational level, daily coffee and tea consumption) and work-related information (i.e., job category, department, weekly working hours, and number of night shifts per month) were collected.

#### 2.3.2 Healthcare worker occupational fatigue scale

The HWOFS comprises 35 items across five dimensions: physical (14 items), cognitive and emotional (7 items), social (4 items), sleep-related (4 items), and auditory fatigue (6 items) (14). Each item was assessed on a 5-point Likert scale (1 = never happened, 5 = always like this), with total scores ranging from 35 to 175 points; higher scores indicate higher levels of occupational fatigue. The HWOFS demonstrated good reliability, with a Cronbach’s alpha coefficient of 0.976 in this study.

#### 2.3.3 Physical workplace comfort perception questionnaire

A self-designed questionnaire assessed HCWs perceptions of the surrounding environment across five dimensions: temperature, humidity, air quality, light, and sound intensity. Each dimension was rated on a five-point Likert scale (1 = comfortable, 5 = intolerable).

### 2.4 Data collection

Data collection was conducted using an online questionnaire administered via the Questionnaire Star platform,^1^ a widely used survey tool in China. The survey link was distributed through the official internal OA system of the hospital between June 10th to 25th, 2025. Participants accessed the questionnaire by scanning a QR code and provided informed consent before proceeding. To improve the quality of the online data collection, each IP address could be used only once to complete the questionnaire, and participants could submit the questionnaire when all options were completed. When the answers provided in a questionnaire were the same or the completion time for the online questionnaire was less than 300 s, the completed questionnaire was rejected. Participants were asked to reflect on their experiences of occupational fatigue over the past month, a period chosen to balance recall accuracy and symptom range. A total of 823 questionnaires were collected. After excluding 89 invalid questionnaires due to completion times under 5 min or uniform responses patterns, 734 valid questionnaires were retained for analysis, yielding an effective response rate of 89.19%.

### 2.5 Statistical analysis

Data analysis was performed using IBM SPSS Statistics (version 26.0) and Mplus (version 8.0). Categorical variables were summarized as frequencies and percentages [*n* (%)]. Normality of continuous variables was assessed using the Kolmogorov–Smirnov test. In this study, the continuous variables deviated significantly from normality (*p* < 0.05); accordingly, they were described as median and interquartile range [M (IQR)]. To ensure comparability across dimensions with different item counts, the item mean score (i.e., dimension sum divided by number of items) was calculated for each of the five HWOFS dimensions. These scores of each dimension were then entered into the LPA. One to five potential profile models were explored sequentially from the initial model (one profile) to the determination of the most appropriate model with a log-likelihood test. The LPA model fit test indices included the Akaike information criterion (AIC), the Bayesian information criterion (BIC), and the adjusted Bayesian information criterion (aBIC), with a lower value indicating a better-fitting model (17). The classification accuracy was evaluated with entropy values (from zero to one, with better values close to one). The Lo–Mendell–Rubin Test (LMR) and bootstrap likelihood ratio test (BLRT) were used to assess the *p* values in the comparisons among models with different numbers of classes. A low *p* value indicated that the k-class model fit better than the k-1-class model. In addition to fit indices, the selection of the optimal profile model was informed by an assessment of competing models based on the posterior classification probabilities of the most likely profile. With probabilities ranging from 0 to 1, the diagonal entries exhibited high values, indicating satisfactory classification accuracy (17). Once the best profile model was determined, differences in categorical variables among fatigue profiles were analyzed using the chi-square test (*χ^2^*) or Fisher’s exact probability. Multiple Logistic regression was performed to identify predictive factors for each profile. Statistical significance was set at a two-tailed *p* value < 0.05.

## 3 Results

### 3.1 Demographic characteristics

Of the 734 participants, 52.18% (*n* = 383) had ≤ 5 years of work experience. A total of 53.68% (*n* = 394) worked 41–50 h per week, and nearly half (*n* = 356, 48.50%) worked 6–10 night shifts per month. More than half reported not consuming excessive amounts of coffee (55.04%) or tea (58.72%). The majority (88.55%) stood or walked for ≤ 8 h per shift. Regarding workload, 64.44% (*n* = 473) reported a moderate workload, and 11.17% (*n* = 82) reported a severe workload. Concerns about continuing in their current position were expressed by 53.54% (*n* = 393) of participants. Detailed information is presented in Table 1.

**Table 1:** Demographic characteristics (*N* = 734).

Characteristics	*n*	%
Gender		
Male	274	37.33
Female	460	62.67
Age (y)		
≤30	321	43.73
31–40	348	47.41
≥41	65	8.86
Educational level		
Associated degree	70	9.54
Bachelor’s degree	400	54.50
Master’s degree	169	23.02
Doctoral degree	95	12.94
BMI(Kg/m^2^)		
≤18.4	60	8.17
18.5–23.9	487	66.35
24–27.9	153	20.84
≥28	34	4.63
Marital status		
Unmarried, divorced, or widowed	326	44.41
Married	408	55.59
Exercise frequency (times/week)		
None	212	28.88
1–2	370	50.41
3–4	120	16.35
≥5	32	4.36
Self-reported health condition		
Poor	39	5.31
Fair	427	58.17
Good	268	36.51
Underlying illness		
Yes	210	28.61
No	524	71.39
Coffee consumption (cups/week)		
None	404	55.04
1–7	291	39.65
≥8	39	5.31
Tea consumption (cups/week)		
None	431	58.72
1–14	269	36.65
≥15	34	4.63
Job category		
Doctor	194	26.43
Nurse	311	42.37
Medical Technicians	61	8.31
Administrative staff	76	10.35
Others	92	12.53
Type of employment		
Formal	538	73.30
Advanced training or standardized residency	99	13.49
Internship	97	13.22
Department		
Surgical ward	209	28.47
Medical ward	132	17.98
Specialized department^*^	158	21.53
Administrative department	74	10.08
Clinical support departments	161	21.93
Working experience (y)		
≤5	383	52.18
6–10	182	24.80
>10	169	23.02
Weekly working hours		
≤40	189	25.75
41–50	394	53.68
>50	151	20.57
Number of night shifts per month		
≤5	212	28.88
6–10	356	48.50
>10	166	22.62
Total daily commuting time (h)		
<1	259	35.29
1–3	449	61.17
>3	26	3.54
Standing or walking hours per shift		
<4	334	45.50
4–8	316	43.05
>8	84	11.44
Learning or training sessions per month		
None	60	8.17
1–2	496	67.57
3–4	136	18.53
≥5	42	5.72
Self-reported workload		
Light	179	24.39
Moderate	473	64.44
Heavy	82	11.17
Intention to stay		
High	320	43.60
With concerns	393	53.54
Low	21	2.86

*Emergency department, intensive care unit and operating room.

### 3.2 Occupational fatigue levels

**Figure 2:**
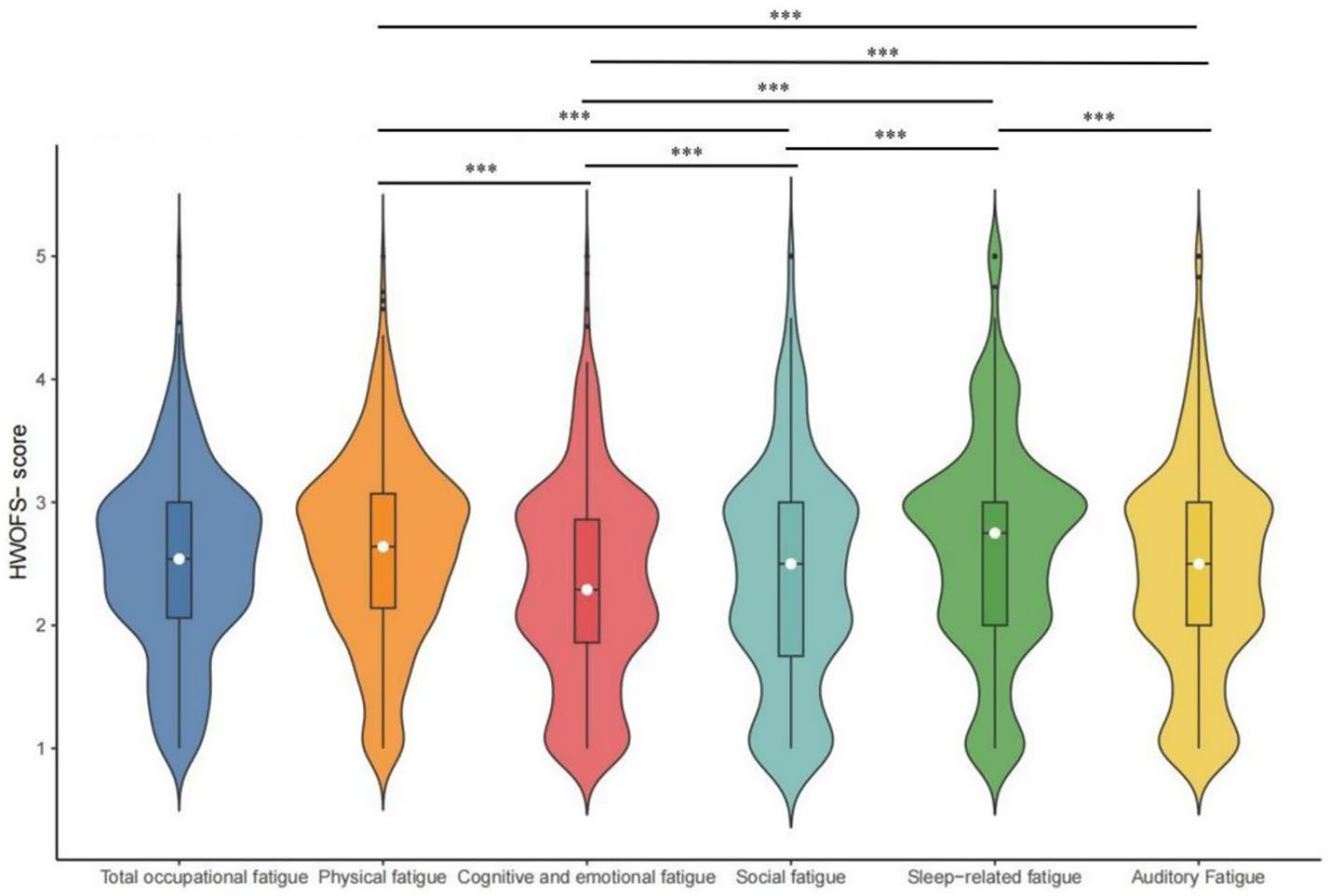
Distribution of total occupational fatigue and five dimensions.

The overall occupational fatigue score was 2.54 (P_25_: 2.06, P_75_: 3.00), indicating a moderate level. Scores for the five dimensions, in descending order, were as follows: sleep-related fatigue, 2.75 (P_25_: 2.00, P_75_: 3.00); physical fatigue, 2.64 (P_25_: 2.14, P_75_: 3.07); auditory fatigue, 2.50 (P_25_: 2.00, P_75_: 3.00); social fatigue, 2.50 (P_25_: 1.75, P_75_: 3.00); and cognitive-emotional fatigue, 2.29 (P_25_: 1.86, P_75_: 2.86). A significant difference among five dimensions was observed (Friedman test, *χ^2^* = 289.174, *p* < 0.005). Distribution patterns varied across dimensions: physical fatigue showed an approximately unimodal distribution, while the other four dimensions exhibited multimodal distributions, suggesting potential subgroup heterogeneity within each of these fatigue domains (Figure 2).

### 3.3 Latent profile analysis

Table 2 presents the fit indices for models with one to five profiles. Although the five-profile model yieled the lowest AIC, BIC, and aBIC values, its smallest profile (*n* = 9, 1.23%) fell below the generally recommended minimum threshold of 5% of the total sample (18, 19), thereby limiting its practical applicability. The four-profile model demonstrated high entropy (>0.800) and the second-lowest AIC, BIC, and aBIC values. The smallest profile of this model comprised 8.86% of the sample. Accordingly, the four-profile model was selected as optimal. The average probabilities for Profiles 1 through 4 in the four-profile model were 98.8, 94.1, 94.8, and 94.4%, respectively, indicating that the LPA solution for the four-profile model possesses good identifiability and reliability.

**Table 2:** Latent profile model fit indicators (*N* = 734).

Profile	AIC	BIC	aBIC	*p*LMR	*p*BLRT	Entropy	Group size for each profile				
							1	2	3	4	5
Profile-1	9069.763	9115.748	9083.994	–	–	–	734				
Profile-2	7353.613	7427.189	7376.383	<0.001	<0.001	0.868	317	417			
Profile-3	6539.044	6640.211	6570.354	0.0026	<0.001	0.890	141	347	246		
Profile-4	5961.667	6090.425	6001.516	<0.001	<0.001	0.914	132	259	278	65	
Profile-5	5832.304	5988.654	5880.692	<0.001	<0.001	0.925	250	132	277	66	9

### 3.4 Characteristics of the four latent profiles

**Figure 3:**
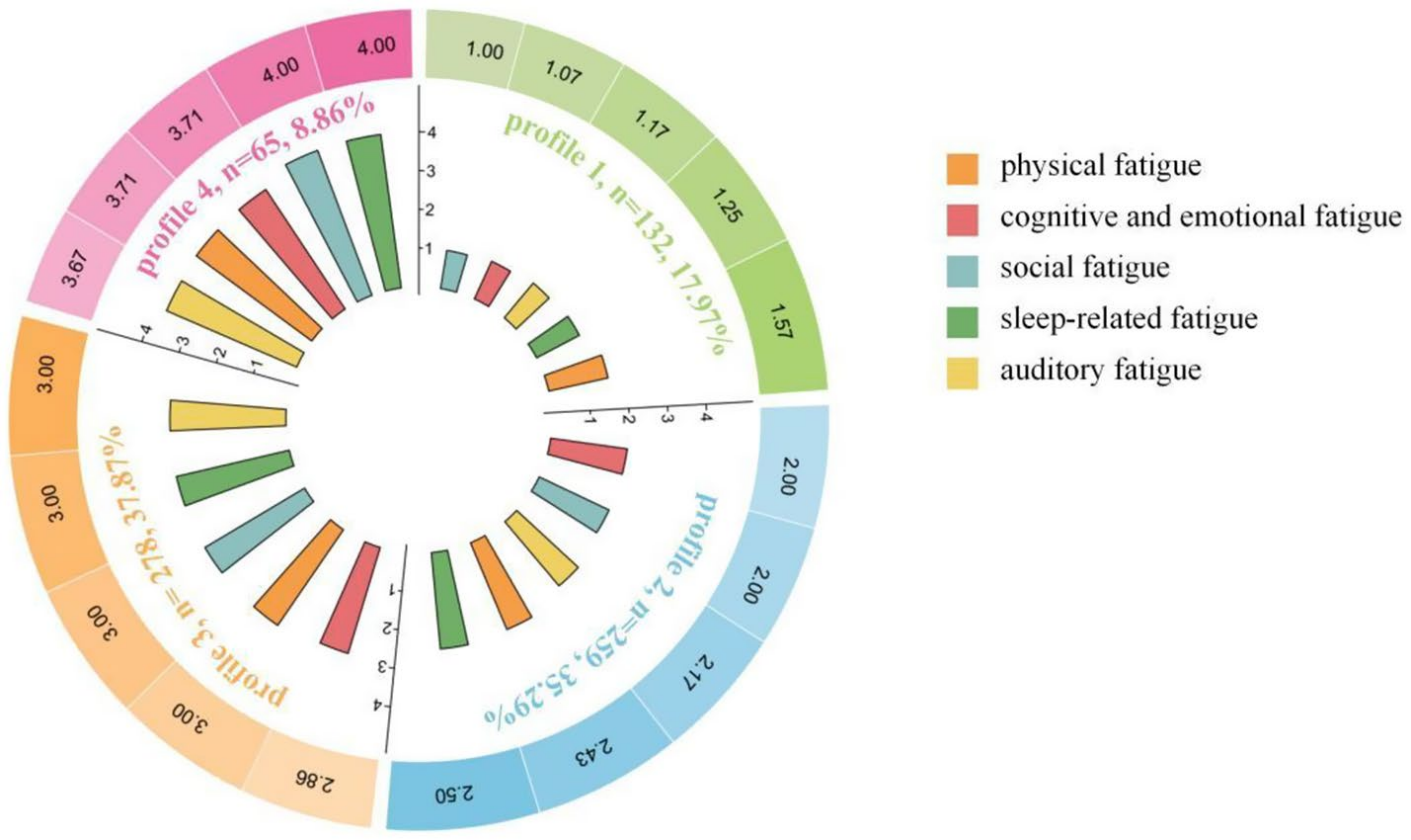
Details of the five dimensions of HWOFS in the four-profile model.

Figure 3 presents the dimension-specific scores of the four latent profiles derived from LPA. The Kruskal-Wallis test indicated statistically significant differences across the four profiles on all five HWOFS dimensions, as reported in the Supplementary Table S1.

Notably, the progressive pattern of these profiles could be interpreted within the chronic stress adaptation continuum, a well-established framework in clinical medicine and occupational health (20). Profile 1 was named as the “Compensated Group” (17.97% of the sample). Participants in this profile exhibited the lowest overall occupational fatigue levels. Scores on all five dimensions of the HWOFS were below the sample mean. This group was characterized by effective compensation against occupational stressors. Profile 2 was named as the “Prodromal-Symptomatic Group” (35.29% of the sample). This profile was characterized by early-stage occupational fatigue, with notably elevated scores on the physical fatigue and sleep-related fatigue. Scores on the other dimensions were around or slightly below the sample mean. Profile 3 was named as the “Decompensated Diffuse Group” (37.87% of the sample). Participants in this profile showed moderate-to-severe occupational fatigue. Scores on all five dimensions exceeded the overall sample means. This profile was distinguished by breakdown of compensatory capacity and diffusion of fatigue symptoms across multiple dimensions. Profile 4 was named the “Systemic Crisis Group” (8.86% of the sample). This profile was characterized by severe and pervasive fatigue across all five dimensions. Scores were substantially higher than those of all other profiles on every dimension, representing a state of exhausted adaptive capacity and systemic functional breakdown.

### 3.5 Univariate analysis across the four latent profiles

As detailed in Table 3, the four profiles differed significantly across nearly all measured variables, indicating marked heterogeneity among the groups. Highly significant differences (all *p* < 0.001) were observed for self-reported workload (*χ^2^* = 249.524), intention to stay (*χ^2^* = 130.944), overall work environment comfort (*χ^2^* = 115.837), and department distribution (*χ^2^* = 97.750). Specifically, a clear gradient emerged across the four profiles: from Profile 1 to Profile 4, the proportion of individuals reporting a “heavy workload” increased from 0.76 to 53.85%, while “high intention to stay” decreased from 74.24 to 18.46%. Similarly, perceived environmental comfort (temperature, humidity, air quality, lighting, and noise) declined noticeably, with the percentage of “comfortable” ratings decreasing from 61.37–85.61% in Profile 1 to 10.77–32.31% in Profile 4 (all *p* < 0.001).

**Table 3:** Inter-profile characteristic differences.

Characteristics		Profile 1	Profile 2	Profile 3	Profile 4	*χ* ^2^	*p*
		*n* (%)	*n* (%)	*n* (%)	*n* (%)		
Gender	Male	62(46.97)	82(31.66)	101(36.33)	29(44.62)	10.395	0.015
	Female	70(53.03)	177(68.34)	177(63.67)	36(55.38)		
Educational level	Associated degree	19(14.39)	25(9.65)	21(7.55)	5(7.69)	23.490	0.005
	Bachelor’s degree	78(59.09)	153(59.07)	142(51.08)	27(41.54)		
	Master’s degree	23(17.42)	55(21.24)	74(26.62)	17(26.15)		
	Doctoral degree	12(9.09)	26(10.04)	41(14.75)	16(24.62)		
BMI(Kg/m^2^)	≤18.4	9(6.82)	24(9.27)	24(8.63)	3(4.62)	23.182	0.006
	18.5–23.9	90(68.18)	173(66.80)	191(68.71)	33(50.77)		
	24–27.9	22(16.67)	50(19.31)	55(19.78)	26(40.00)		
	≥28	11(8.33)	12(4.63)	8(2.88)	3(4.62)		
Exercise frequency (times/week)	None	27(20.45)	73(28.19)	85(30.58)	27(41.54)	20.752	0.014
	1–2	64(48.48)	141(54.44)	138(49.64)	27(41.54)		
	3–4	35(26.52)	36(13.90)	40(14.39)	9(13.85)		
	≥5	6(4.55)	9(3.47)	15(5.40)	2(3.08)		
Self-reported health condition	Poor	2(1.52)	7(2.70)	20(7.19)	10(15.38)	56.933	< 0.001
	Fair	60(45.45)	140(54.05)	182(65.47)	45(69.23)		
	Good	70(53.03)	112(43.24)	76(27.34)	10(15.38)		
Underlying diseases	Yes	19(14.39)	68(26.25)	93(33.45)	30(46.15)	26.752	< 0.001
	No	113(85.61)	191(73.75)	185(66.55)	35(53.85)		
Coffee consumption (cups/week)	None	79(59.85)	150(57.92)	147(52.88)	28(43.08)	15.661	0.016
	1–7	49(37.12)	100(38.61)	114(41.01)	28(43.08)		
	≥8	4(3.03)	9(3.47)	17(6.12)	9(13.85)		
Job category	Doctor	22(16.67)	53(20.46)	88(31.65)	31(47.69)	73.102	< 0.001
	Nurse	45(34.09)	123(47.49)	121(43.53)	22(33.85)		
	Medical Technicians	9(6.82)	27(10.42)	23(8.27)	2(3.08)		
	Administrative staff	18(13.64)	27(10.42)	29(10.43)	2(3.08)		
	Others	38(28.79)	29(11.20)	17(6.12)	8(12.31)		
Type of employment	Formal	81(61.36)	206(79.54)	211(75.90)	40(61.54)	35.387	< 0.001
	Advanced training or standardized residency	36(27.27)	20(7.72)	31(11.15)	12(18.46)		
	Internship	15(11.36)	33(12.74)	36(12.95)	13(20.00)		
Department	Surgical ward	27(20.45)	71(27.41)	83(29.86)	28(43.08)	97.750	< 0.001
	Medical ward	15(11.36)	46(17.76)	57(20.50)	14(21.54)		
	Specialized department	11(8.33)	52(20.08)	80(28.78)	15(23.08)		
	Administrative department	18(13.64)	22(8.49)	31(11.15)	3(4.62)		
	Other auxiliary departments	61(46.21)	68(26.25)	27(9.71%)	5(7.69%)		
Weekly working hours	≤40	44(33.33)	61(23.55)	75(26.98)	9(13.85)	32.882	< 0.001
	41–50	71(53.79)	158(61.00)	135(48.56)	30(46.15)		
	>50	17(12.88)	40(15.44)	68(24.46)	26(40.00)		
Number of night shifts per month	≤5	59(44.70)	65(25.10)	75(26.98)	13(20.00)	26.027	< 0.001
	6–10	52(39.39)	140(54.05)	134(48.20)	30(46.15)		
	>10	21(15.91)	54(20.85)	69(24.82)	22(33.85)		
Total daily commuting time (h)	<1	43(32.58)	102(39.38)	97(34.89)	17(26.15)	12.852	0.045
	1–3	82(62.12)	152(58.69)	173(62.23)	42(64.62)		
	>3	7(5.30)	5(1.93)	8(2.88)	6(9.23)		
Standing or walking hours per shift	<4	79(59.85)	115(44.40)	118(42.45)	22(33.85)	22.626	0.001
	4–8	42(31.82)	118(45.56)	128(46.04)	28(43.08)		
	>8	11(8.33)	26(10.04)	32(11.51)	15(23.08)		
Learning or training sessions per month	None	10(7.58)	15(5.79)	27(9.71)	8(12.31)	21.973	0.009
	1–2	91(68.94)	185(71.43)	181(65.11)	39(60.00)		
	3–4	20(15.15)	53(20.46)	54(19.42)	9(13.85)		
	≥5	11(8.33)	6(2.32)	16(5.76)	9(13.85)		
Self-reported workload	Light	78(59.09)	58(22.39)	38(13.67)	5(7.69)	249.524	< 0.001
	Moderate	53(40.15)	194(74.90)	201(72.30)	25(38.46)		
	Heavy	1(0.76)	7(2.70)	39(14.03)	35(53.85)		
Intention to stay	High	98(74.24)	135(52.12)	75(26.98)	12(18.46)	130.944	< 0.001
	With concerns	33(25.00)	121(46.72)	195(70.14)	44(67.69)		
	Low	1(0.76)	3(1.16)	8(2.88)	9(13.85)		
Ambient temperature at the workplace	Comfortable	113(85.61)	188(72.59)	167(60.07)	21(32.31)	65.318	< 0.001
	Unomfortable	19(14.39)	71(27.41)	111(39.93)	44(67.69)		
Ambient humidity at the workplace	Comfortable	113(85.61)	183(70.66)	161(57.91)	20(30.77)	67.882	< 0.001
	Unomfortable	19(14.39)	76(29.34)	117(42.09)	45(69.23)		
Air quality at the workplace	Comfortable	92(69.70)	122(47.10)	90(32.37)	11(16.92)	71.037	< 0.001
	Unomfortable	40(30.30)	137(52.90)	188(67.63)	54(83.08)		
Lighting intensity at the workplace	Comfortable	102(77.27)	161(62.16)	110(39.57)	17(26.15)	78.909	< 0.001
	Unomfortable	30(22.73)	98(37.84)	168(60.43)	48(73.85)		
Sound intensity at the workplace	Comfortable	81(61.37)	114(44.02)	67(24.10)	7(10.77)	78.385	< 0.001
	Unomfortable	51(38.63)	145(55.98)	211(75.90)	58(89.23)		
Overall work environment	Comfortable	100(75.76)	146(43.63)	82(29.50)	9(13.85)	115.837	< 0.001
	Unomfortable	32(24.24)	113(56.37)	196(70.50)	56(86.15)		

### 3.6 Multivariable analysis across the four latent profiles

**Figure 4:**
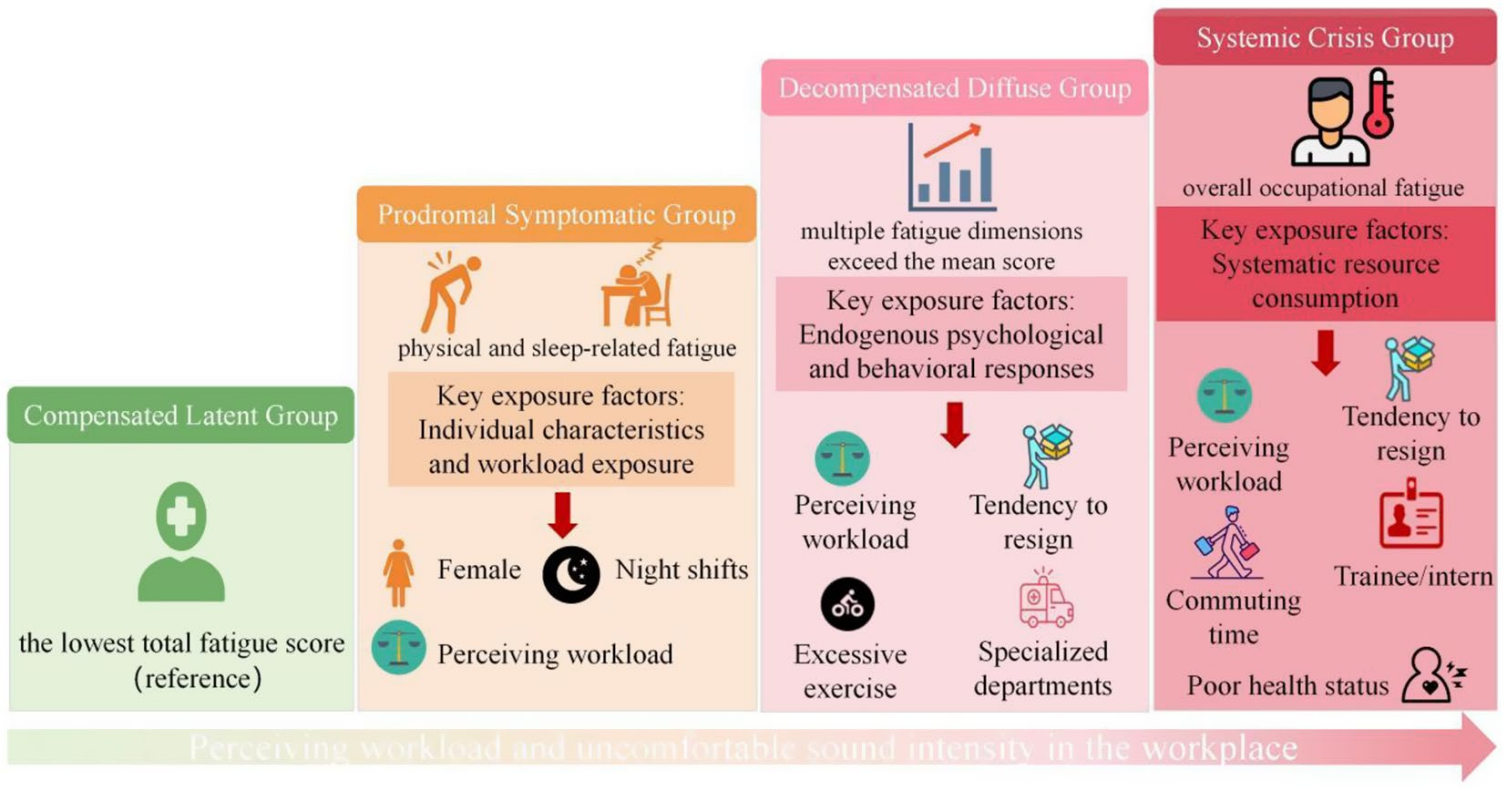
The portrait of HCWs occupational fatigue.

Multiple logistic regression analysis was performed using the four occupational fatigue profiles as the dependent variables, with the “Compensated Group” as the reference. Variables that were statistically significant in the univariate analysis were included as independent variables. The results are summarized in Figure 4. Multicollinearity diagnostics and confidence intervals for the odds ratios are provided in Supplementary Tables S2, S3, respectively.

Compared with the “Compensated Group,” the odds of being in the “Prodromal-Symptomatic Group” were significantly higher among females (OR = 1.961, *p* = 0.038), those working 6–10 night shifts per month (OR = 2.752, *p* = 0.002), and those perceiving a moderate workload (OR = 3.654, *p* < 0.001). Conversely, significantly lower odds were observed among staff working in administrative departments (OR = 0.208, *p* = 0.048) or other clinical support departments (OR = 0.340, *p* = 0.026), those participating in training sessions 1–2 times (OR = 0.301, *p* = 0.040) or ≥5 times per month (OR = 0.052, *p* = 0.001), and those reporting a comfortable acoustic environment at work (OR = 0.480, *p* = 0.036).

Compared with the “Compensated Group,” the odds of belonging to the “Decompensated Diffuse Group” were significantly elevated for staff who exercised ≥5 times per week (OR = 5.583, *p* = 0.022), worked in specialized departments (OR = 4.281, *p* = 0.004), perceived a moderate (OR = 4.212, *p* < 0.001) or severe workload (OR = 91.076, *p* = 0.003), and expressed concerns regarding their intention to stay in their current role (OR = 4.306, *p* < 0.001). Conversely, significantly lower odds were observed among staff with a BMI ≥ 28 kg/m^2^ (OR = 0.203, *p* = 0.028), those working in other clinical support departments (OR = 0.053, *p* < 0.001), those participating in training sessions 1–2 times (OR = 0.150, *p* = 0.002) or ≥5 times per month (OR = 0.048, *p* = 0.001), and those reporting a comfortable acoustic environment (OR = 0.188, *p* < 0.001).

Compared with the “Compensated Group,” staff in the “Systemic Crisis Group” had significantly higher odds of being resident physician/trainee (OR = 5.673, *p* = 0.012) or an intern (OR = 5.781, *p* = 0.029); working in specialized departments (OR = 4.260, *p* = 0.035); commuting more than 3 h per day (OR = 20.496, *p* = 0.003); perceiving a moderate (OR = 3.909, *p* = 0.050) or, most markedly, a severe workload (OR = 788.152, *p* < 0.001); and expressing concerns about (OR = 3.519, *p* = 0.019) or having a low intention to stay (OR = 45.096, *p* = 0.019). Conversely, significantly protective associations were observed for staff reporting fair (OR = 0.083, *p* = 0.025) or good health status (OR = 0.017, *p* = 0.001), those working in other clinical support departments (OR = 0.026, *p* < 0.001), participating in training sessions 1–2 (OR = 0.109, *p* = 0.008) or 3–4 times per month (OR = 0.096, *p* = 0.015), and reporting a comfortable workplace acoustic environment at work (OR = 0.141, *p* = 0.015).

## 4 Discussion

A cohort of 734 HCWs from a Chinese tertiary grade A hospital was assessed using a validated occupational fatigue scale. Analysis revealed multidimensional characteristics and significant heterogeneity in HCWs’ occupational fatigue. Overall, participants reported moderate fatigue, highest in sleep-related fatigue and lowest in cognitive-emotional fatigue. Latent profile analysis delineated the population into four distinct fatigue profiles: “Compensated Group” (17.97%), “Prodromal-Symptomatic Group” (35.29%), “Decompensated Diffuse Group” (37.87%), and “Systemic Crisis Group” (8.86%). These profiles suggest that a considerable proportion of staff endure severe fatigue, either confined to specific dimensions or pervasive across multiple domains, highlighting the need for precise screening and stratified management strategies. Multiple logistic regression further identified key predictors of subgroup membership, including gender, workload, night-shift frequency, training participation, departmental characteristics, and acoustic environmental comfort. Collectively, the results corroborate the complex, multilevel structure of occupational fatigue in hospital settings, and clarify its interplay with environmental, organizational, and individual factors. This evidence supports the development of targeted, effective intervention strategies.

### 4.1 Characteristics of occupational fatigue among HCWs

The study sample revealed four heterogeneous subgroups of occupational fatigue. This four-class model diverges from the conventional three-profile structures commonly identified in prior research. Previously, studies often conceptualized HCWs’ fatigue within a tripartite framework, for instance, distinguishing between “low-fatigue/high-recovery group,” “moderate-fatigue/moderate-recovery group,” and “high-fatigue/low-recovery group” (21), or classifying patterns as “chronic fatigue, acute fatigue, and inter-shift recovery” (22). These profiles describe the balance between fatigue accumulation and recuperation but do not adequately capture the multidimensional nature of fatigue. This study employed the HWOFS (encompassing physical, cognitive-emotional, social, sleep-related, and auditory fatigue). The resulting four-profile solution extends beyond a simple severity continuum (low to high) by characterizing three distinct pathological progression states: prodromal symptoms, decompensated diffuse, and systemic crisis. This structure implies that fatigue development may involve a critical tipping point beyond which symptoms transition from being limited and prodromal to becoming widespread and multisystemic.

The four latent profiles identified in this study represented a progressive spectrum of fatigue severity and dysregulation. The “Compensated Group” partially aligns with the classic “low-fatigue/high-recovery” phenotype described in earlier research (21). Its defining feature was consistently low fatigue scores across all dimensions, reflecting intact compensatory resources. The “Prodromal-Symptomatic Group” manifests early yet discernible fatigue, predominantly within the physical and sleep-related dimensions. This manifestation can be primarily attributed to the strenuous physical demands characteristic of hospital settings, including activities requiring prolonged orthostatic posture, constant ambulation, and the circadian disruption associated with night shift work (23). In the “Decompensated Diffuse Group”, fatigue symptoms transcended the physical and sleep domains, diffusing significantly into cognitive-emotional, social, and auditory dimensions. Critically, scores across all dimensions surpassed the sample mean, signaling the impending failure of compensatory mechanisms. This reflects a key transition wherein fatigue generalizes from a predominantly physiological state to one that impairs psychological and social functioning. This critical shift, often obscured in studies using aggregate fatigue scores, was distinctly captured through the multidimensional HWOFS and latent profile analysis. Representing the most severe fatigue spectrum, the “Systemic Crisis Group” was marked by uniformly high fatigue scores across all dimensions, indicative of a potentially exhausted regulatory system. Despite its relatively small size, this subgroup demonstrated the most severe functional impairment and showed a robust clinical association with occupational burnout and psychosomatic disorders. Its identification highlights an urgent clinical priority: the timely detection of these high-risk individuals within the healthcare workforce and the implementation of immediate, multifaceted interventions to prevent further deterioration.

Overall, the four-profile solution demonstrated favorable statistical properties. The entropy value was 0.914, exceeding the recommended threshold of 0.80, and the average posterior probabilities for the four profiles ranged from 0.941 to 0.988, indicating high classification certainty (17). The smallest profile (Systemic crisis) comprised 8.86% of the sample, above the minimum class size of 5% suggested in previous studies (18, 19), but external validation is still needed.

### 4.2 Exposure factors associated with occupational fatigue among HCWs

The predictive factors for membership in the “Prodromal-Symptomatic Group” were predominantly associated with workload exposure. First, individuals perceiving a moderate workload had a 3.654 times higher risk of belonging to this group than those perceiving a mild workload, indicating that subjective stress experience constitutes a primary source of early fatigue signals. This aligns with a study conducted in Iran (24), which reported a strong positive correlation between workload and physical fatigue. Second, circadian rhythm disruption induced by the shift system significantly increased risk. Staff working 6–10 night shifts per month had a 2.752 times higher risk of inclusion in this group than those working fewer than 5 night shifts. Night-shift work disrupts circadian rhythms and impairs the sleep–wake cycle, forcing HCWs to remain awake during biological night periods while hindering full recovery during rest periods (7, 25). This leads to cumulative sleep deprivation, circadian misalignment, and fatigue. Furthermore, gender exerted a significant influence, with women being 1.961 times more likely than men to enter the “Prodromal-Symptomatic Group.” This is consistent with most existing researches (26, 27). A previous study has shown that, compared with men, women often reported neck, shoulders, waist, or back pain (28). From a physiological viewpoint, gender-related differences could result from estrogen and progesterone (the major female sex hormones) (28). In addition, staff in administrative departments (OR = 0.208) and other clinical support departments (OR = 0.340) had significantly lower risk than those in surgical departments, reflecting variations in the nature of work across departments. In contrast to the high-exertion environment of clinical frontline departments, roles within administrative and support departments are generally characterized by reduced physical demands, regular working hours, and limited direct patient care responsibilities. These conditions collectively contribute to a lower incidence and severity of both physical and sleep fatigue among staff in these areas.

Analysis of the “Decompensated Diffuse Group” indicated that advancing fatigue engages a broader set of predictors, encompassing personal health behaviors and organizational psychological factors beyond workload. A sustained workload pressure is central to this process. Perceiving a moderate or heavy workload increased the risk of belonging to this group by 4.212 and 91.076 times, respectively, compared to perceiving a mild workload. This finding corresponds with recent evidence reporting workload coefficients of 5.70–6.76 for acute fatigue and 6.71–7.16 for chronic fatigue (29), confirming that excessive workload is the primary driver of fatigue diffusion across dimensions. Environmental and departmental determinants further shape risk. Personnel in specialized settings (the emergency room, intensive care unit, or operating room) face a 4.281-fold higher risk than those in surgical wards, due to intense decision-making demands, emotional load, and time–pressure (4, 30). Otherwise, psychological resource depletion appears to mediate this progression: individuals with low retention willingness exhibited a 4.306 times higher risk, suggesting reciprocal deterioration between eroding professional identity and spreading fatigue. A counterintuitive finding emerged regarding physical activity: exercising five or more times per week elevated the risk (OR = 5.583), contrary to conventional protective associations (31). This may reflect a “reverse compensatory” pattern, where intense exercise is used to mitigate existing fatigue, though it may also indicate underlying high physiological stress. Alternatively, this association may reflect elevated physiological stress prior to exercise, or residual confounding. Future longitudinal studies are needed to clarify the temporal direction.

The analysis of the “Systemic Crisis Group” confirmed that working in specialized departments, perceived workload, and retention intention continued to predict profile membership. Among these, the perception of heavy workload and a marked decline in retention intention emerged as dominant determinants. Additionally, early-career personnel, trainees, and interns faced markedly elevated risks (ORs = 5.673 and 5.781, respectively), highlighting the vulnerability associated with professional instability and limited institutional support (32). Notably, a daily commute longer than 3 hrs exerted an extraordinary risk effect (OR = 20.496). This result is consistent with the findings of previous studies that explored the relationship between commuting time and burnout (33, 34). This relationship can be understood through the lens of chronic stress: prolonged commuting acts as a significant non-work demand that depletes time and psychological resources, impairs work-life balance, and induces emotional exhaustion, cumulatively contributing to severe occupational fatigue.

Further supporting the role of modifiable factors, this study identified self-reported good health condition, a comfortable acoustic environment, and training participation as significant protective elements. These findings align with established literature. Notably, a large-scale study of 3,818 U. S. nurses linked high noise exposure to worse health outcomes, increased stress, greater burnout (reflected in lower burnout scores), and diminished professional quality of life (35). The pathogenic pathway likely involves noise as a stressor that dysregulates the autonomic nervous system, thereby prolonging sleep onset, reducing total sleep time, and ultimately propagating daytime fatigue and cognitive-functional deficits (36).

Notably, a few methodological caveats pertain to the multinomial logistic regression. Variables such as workload and intention to stay yielded extreme odds ratios, particularly when comparing Profile 4 with Profile 1. These extreme values reflect statistical instability resulting from sparse data and near-complete separation rather than clinically meaningful effect sizes. Future studies with larger, more balanced samples are needed to obtain more stable estimates.

### 4.3 Limitations

This study had some limitations. First, with respect to sample representativeness, data were collected from a single tertiary hospital, potentially limiting the generalizability of the findings. Moreover, the exclusion criteria may have introduced selection and survivorship bias: only currently employed HCWs were included, whereas those who had left the profession due to extreme fatigue were not captured. Consequently, the prevalence of more severe fatigue profiles may be underestimated in the present results. Second, the data relied on self-report, a method prone to recall and social desirability bias. Hence, future studies should consider incorporating multi-method approaches (e.g., actigraphy, physiological monitoring, ecological momentary assessment) to obtain more objective measurements. Beyond these methodological constraints, future research could explore integrating Building Information Modeling (BIM) with real-time environmental sensors to objectively quantify the impact of environmental exposures on fatigue.

## 5 Conclusion

This study used latent profile analysis to identify four distinct occupational fatigue profiles among HCWs, demonstrating that fatigue manifests as discrete subgroups with varying severity and characteristics rather than as a uniform condition. Acoustic environment, perceived workload, and departmental affiliation were significant determinants of profile membership. A dual-pronged strategy is recommended: optimizing the physical work environment (particularly acoustic comfort) and implementing profile-specific interventions. This targeted approach can effectively mitigate fatigue, preserve human resources, and enhance both healthcare workers’ well-being and the quality of patient care.

## Data Availability

The raw data supporting the conclusions of this article will be made available by the authors, without undue reservation.

## Footnotes

1 Wenjuanxing, https://www.wjx.cn/
