# Supplementary material for: Multiple exposure sources and occupational fatigue profiles among healthcare workers: a cross-sectional latent profile analysis
Source: Front Public Health. 2026 May 20;14:1811642. doi: 10.3389/fpubh.2026.1811642 (PMC13230211; doi:10.3389/fpubh.2026.1811642)
Supplement: Supplementary file 1 [file Table_1.docx]

**Supplementary Material**

Table S1. Scores on the five dimensions of HWOFS across four profiles (*N*=734), [M (IQR)].

|  | Profile 1 (*n*=132) | Profile 2 (*n*=259) | Profile 3 (*n*=278) | Profile 4 (*n*=65) | *H* | *P* |
| --- | --- | --- | --- | --- | --- | --- |
| Physical fatigue | 1.57  (1.07,1.79) | 2.43  (2.14,2.64) | 3.00  (2.86,3.21) | 3.71  (3.50,4.04) | 533.484 | < 0.001 |
| Cognitive and emotional fatigue | 1.07  (1.000,1.286) | 2.00  (1.86,2.29) | 2.86  (2.57,3.00) | 3.71  (3.50,4.00) | 605.736 | < 0.001 |
| Social fatigue | 1.00  (1.00,1.25) | 2.00  (2.00,2.50) | 3.00  (2.50,3.00) | 4.00  (3.50,4.13) | 527.524 | < 0.001 |
| Sleep-related fatigue | 1.25  (1.00,1.75) | 2.50  (2.00,2.75) | 3.00  (2.75,3.25) | 4.00  (3.75,4.38) | 469.416 | < 0.001 |
| Auditory fatigue | 1.17  (1.00,1.50) | 2.17  (2.00,2.50) | 3.00  (2.67,3.17) | 3.67  (3.08,4.08) | 488.536 | < 0.001 |

Note: M, Median; IQR, Interquartile range; *H*, Kruskal-Wallis test statistic.

Table S2. Results of multicollinearity diagnostics of independent variables.

| Variable | VIF | Tolerance |
| --- | --- | --- |
| Gender（ref: Male） | | |
| Female | 1.487 | 0.672 |
| Educational level（ref: Associated degree） | | |
| Bachelor’s degree | 3.650 | 0.274 |
| Master’s degree | 3.695 | 0.271 |
| Doctoral degree | 3.210 | 0.312 |
| BMI（ref: 18.5–23.9, Kg/m^2^） | | |
| ≤18.4 | 1.127 | 0.887 |
| 24–27.9 | 1.202 | 0.832 |
| ≥28 | 1.161 | 0.862 |
| Exercise frequency（ref: None, times/week） | | |
| 1–2 | 1.526 | 0.655 |
| 3–4 | 1.600 | 0.625 |
| ≥5 | 1.266 | 0.790 |
| Self-reported health condition（ref: Poor） | | |
| Fair | 5.499 | 0.182 |
| Good | 6.011 | 0.166 |
| Underlying diseases（ref: Yes） | | |
| No | 1.203 | 0.831 |
| Coffee consumption（ref: None, cups/week） | | |
| 1–7 | 1.129 | 0.885 |
| ≥8 | 1.141 | 0.876 |
| Job category（ref: Administrative staff） | | |
| Doctor | 10.131 | 0.099 |
| Nurse | 12.065 | 0.083 |
| Medical Technicians | 3.734 | 0.268 |
| Others | 4.579 | 0.218 |
| Department（ref: Surgical ward） | | |
| Medical ward | 1.470 | 0.680 |
| Specialized department | 1.587 | 0.630 |
| Administrative department | 4.424 | 0.226 |
| Other auxiliary departments | 2.874 | 0.348 |
| Type of employment（ref: Formal） | | |
| Advanced training or standardized residency | 1.370 | 0.730 |
| Internship | 1.560 | 0.641 |
| Weekly working hours（ref: ≤40） | | |
| 41-50 | 1.635 | 0.612 |
| ＞50 | 2.087 | 0.479 |
| Number of night shifts per month（ref: ≤5） | | |
| 6–10 | 1.677 | 0.596 |
| >10 | 1.858 | 0.538 |
| Total daily commuting time（ref: <1, h） | | |
| 1–3 | 1.169 | 0.856 |
| >3 | 1.185 | 0.844 |
| Standing or walking hours per shift（ref: <4, h） | | |
| 4–8 | 1.634 | 0.612 |
| >8 | 1.450 | 0.690 |
| Learning or training sessions per month（ref: None） | | |
| 1–2 | 3.492 | 0.286 |
| 3–4 | 2.999 | 0.333 |
| ≥5 | 1.800 | 0.556 |
| Self-reported workload（ref: Light） | | |
| Moderate | 1.826 | 0.547 |
| Heavy | 2.067 | 0.484 |
| Intention to stay（ref: High） | | |
| With concerns | 1.333 | 0.750 |
| Low | 1.189 | 0.841 |
| Physical workplace comfort perception（ref: Comfortable） | | |
| Temperature | 2.198 | 0.455 |
| Humidity | 2.262 | 0.442 |
| Air quality | 2.265 | 0.441 |
| Lighting intensity | 1.970 | 0.508 |
| Sound intensity | 1.986 | 0.504 |
| Overall work environment | 2.894 | 0.346 |

Table S3. Multivariable Analysis Across the Four Latent Profiles

| Variable | Profile 2 (vs. Profile 1) | | | | |  | Profile 3 (vs. Profile 1) | | | | |  | Profile 4 (vs. Profile 1) | | | | |
| --- | --- | --- | --- | --- | --- | --- | --- | --- | --- | --- | --- | --- | --- | --- | --- | --- | --- |
|  | *B* | *SE* | *OR* | *95%CI* | *P* |  | *B* | *SE* | *OR* | *95%CI* | *P* |  | *B* | *SE* | *OR* | *95%CI* | *P* |
| Gender（ref: Male） | | | | | |  |  |  |  |  |  |  |  |  |  |  |  |
| Female | **0.673** | **0.324** | **1.961** | **1.039–3.700** | **0.038** |  | 0.670 | 0.361 | 1.954 | 0.963–3.963 | 0.063 |  | 0.748 | 0.555 | 2.112 | 0.711–6.273 | 0.178 |
| Educational level（ref: Associated degree） | | | | | |  |  |  |  |  |  |  |  |  |  |  |  |
| Bachelor’s degree | -0.616 | 0.447 | 0.540 | 0.225–1.296 | 0.168 |  | -0.630 | 0.524 | 0.532 | 0.191–1.487 | 0.229 |  | -0.471 | 0.845 | 0.624 | 0.119–3.272 | 0.577 |
| Master’s degree | -0.735 | 0.554 | 0.479 | 0.162–1.420 | 0.184 |  | -0.536 | 0.635 | 0.585 | 0.169–2.029 | 0.398 |  | 0.471 | 1.038 | 1.601 | 0.209–12.251 | 0.650 |
| Doctoral degree | -1.210 | 0.687 | 0.298 | 0.078–1.145 | 0.078 |  | -0.850 | 0.768 | 0.427 | 0.095–1.924 | 0.268 |  | 0.413 | 1.181 | 1.511 | 0.149–15.305 | 0.727 |
| BMI（ref: 18.5–23.9, Kg/m^2^） | | | | | |  |  |  |  |  |  |  |  |  |  |  |  |
| ≤18.4 | 0.880 | 0.543 | 2.412 | 0.832–6.990 | 0.105 |  | 0.905 | 0.593 | 2.472 | 0.773–7.903 | 0.127 |  | 1.015 | 0.889 | 2.759 | 0.483–15.771 | 0.254 |
| 24–27.9 | 0.110 | 0.378 | 1.116 | 0.532–2.343 | 0.771 |  | -0.053 | 0.409 | 0.948 | 0.425–2.113 | 0.896 |  | 0.863 | 0.560 | 2.371 | 0.792–7.101 | 0.123 |
| ≥28 | -0.421 | 0.579 | 0.656 | 0.211–2.043 | 0.467 |  | **-1.595** | **0.727** | **0.203** | **0.049–0.844** | **0.028** |  | -2.206 | 1.239 | 0.110 | 0.010–1.249 | 0.075 |
| Exercise frequency（ref: None, times/week） | | | | | |  |  |  |  |  |  |  |  |  |  |  |  |
| 1–2 | -0.068 | 0.340 | 0.934 | 0.480–1.819 | 0.841 |  | -0.021 | 0.371 | 0.979 | 0.473–2.028 | 0.955 |  | -0.161 | 0.547 | 0.851 | 0.292–2.486 | 0.768 |
| 3–4 | -0.741 | 0.441 | 0.477 | 0.201–1.133 | 0.093 |  | -0.218 | 0.492 | 0.804 | 0.307–2.110 | 0.658 |  | -0.214 | 0.756 | 0.807 | 0.183–3.551 | 0.777 |
| ≥5 | 0.256 | 0.725 | 1.292 | 0.312–5.353 | 0.724 |  | **1.720** | **0.753** | **5.583** | **1.276–24.421** | **0.022** |  | 1.354 | 1.279 | 3.875 | 0.316–47.518 | 0.290 |
| Self-reported health condition（ref: Poor） | | | | | |  |  |  |  |  |  |  |  |  |  |  |  |
| Fair | -0.376 | 0.975 | 0.687 | 0.102–4.638 | 0.700 |  | -1.243 | 0.990 | 0.288 | 0.041–2.007 | 0.209 |  | **-2.493** | **1.115** | **0.083** | **0.009–0.735** | **0.025** |
| Good | -0.272 | 1.005 | 0.762 | 0.106–5.464 | 0.787 |  | -1.845 | 1.028 | 0.158 | 0.021–1.185 | 0.073 |  | **-4.101** | **1.234** | **0.017** | **0.001–0.186** | **0.001** |
| Underlying diseases（ref: Yes） | | | | | |  |  |  |  |  |  |  |  |  |  |  |  |
| No | -0.283 | 0.364 | 0.753 | 0.369–1.539 | 0.437 |  | -0.595 | 0.388 | 0.551 | 0.258–1.179 | 0.125 |  | -1.031 | 0.529 | 0.357 | 0.126–1.007 | 0.051 |
| Coffee consumption（ref: None, cups/week） | | | | | |  |  |  |  |  |  |  |  |  |  |  |  |
| 1–7 | -0.014 | 0.281 | 0.986 | 0.569–1.710 | 0.961 |  | 0.117 | 0.310 | 1.124 | 0.612–2.063 | 0.707 |  | 0.272 | 0.485 | 1.312 | 0.507–3.398 | 0.576 |
| ≥8 | 0.130 | 0.721 | 1.138 | 0.277–4.681 | 0.857 |  | 0.821 | 0.733 | 2.272 | 0.540–9.564 | 0.263 |  | 1.636 | 0.958 | 5.135 | 0.785–33.568 | 0.088 |
| Job category（ref: Administrative staff） | | | | | |  |  |  |  |  |  |  |  |  |  |  |  |
| Doctor | -0.592 | 0.804 | 0.553 | 0.114–2.673 | 0.461 |  | -0.694 | 1.032 | 0.500 | 0.066–3.779 | 0.501 |  | 0.372 | 1.798 | 1.451 | 0.043–49.197 | 0.836 |
| Nurse | -1.256 | 0.748 | 0.285 | 0.066–1.235 | 0.093 |  | -1.450 | 0.991 | 0.235 | 0.034–1.636 | 0.143 |  | 0.138 | 1.801 | 1.148 | 0.034–39.170 | 0.939 |
| Medical Technicians | 0.094 | 0.819 | 1.098 | 0.221–5.470 | 0.909 |  | 0.701 | 1.043 | 2.016 | 0.261–15.562 | 0.501 |  | 0.073 | 1.922 | 1.076 | 0.025–46.500 | 0.970 |
| Others | -0.674 | 0.691 | 0.509 | 0.132–1.973 | 0.329 |  | -0.556 | 0.914 | 0.574 | 0.096–3.440 | 0.543 |  | 1.815 | 1.642 | 6.140 | 0.246–153.536 | 0.269 |
| Department（ref: Surgical ward） | | | | | |  |  |  |  |  |  |  |  |  |  |  |  |
| Medical ward | 0.259 | 0.438 | 1.296 | 0.549–3.055 | 0.554 |  | 0.548 | 0.463 | 1.730 | 0.698–4.287 | 0.236 |  | 0.720 | 0.665 | 2.054 | 0.558–7.559 | 0.279 |
| Specialized department | 0.773 | 0.491 | 2.165 | 0.827–5.667 | 0.115 |  | **1.454** | **0.511** | **4.281** | **1.572–11.657** | **0.004** |  | **1.449** | **0.689** | **4.260** | **1.104–16.433** | **0.035** |
| Administrative department | **-1.570** | **0.796** | **0.208** | **0.044–0.990** | **0.048** |  | -1.717 | 0.998 | 0.180 | 0.025–1.272 | 0.086 |  | -1.669 | 1.597 | 0.188 | 0.008–4.309 | 0.296 |
| Other auxiliary departments | **-1.079** | **0.483** | **0.340** | **0.132–0.877** | **0.026** |  | **-2.947** | **0.618** | **0.053** | **0.016–0.176** | **<0.001** |  | **-3.638** | **0.996** | **0.026** | **0.004–0.185** | **<0.001** |
| Type of employment（ref: Formal） | | | | | |  |  |  |  |  |  |  |  |  |  |  |  |
| Advanced training or standardized residency | -1.222 | 0.401 | 0.295 | 0.134–0.647 | 2.000 |  | -0.165 | 0.443 | 0.848 | 0.356–2.019 | 0.709 |  | **1.736** | **0.692** | **5.673** | **1.460–22.044** | **0.012** |
| Internship | 0.135 | 0.473 | 1.145 | 0.453–2.891 | 0.775 |  | 0.345 | 0.545 | 1.412 | 0.486–4.107 | 0.526 |  | **1.755** | **0.805** | **5.781** | **1.192–28.030** | **0.029** |
| Weekly working hours（ref: ≤40） | | | | | |  |  |  |  |  |  |  |  |  |  |  |  |
| 41-50 | 0.020 | 0.319 | 1.021 | 0.546–1.909 | 0.949 |  | -0.509 | 0.355 | 0.601 | 0.300–1.206 | 0.152 |  | 0.278 | 0.601 | 1.320 | 0.407–4.285 | 0.644 |
| ＞50 | 0.264 | 0.495 | 1.302 | 0.494–3.433 | 0.594 |  | -0.207 | 0.530 | 0.813 | 0.288–2.298 | 0.696 |  | -0.145 | 0.775 | 0.865 | 0.190–3.947 | 0.851 |
| Number of night shifts per month（ref: ≤5） | | | | | |  |  |  |  |  |  |  |  |  |  |  |  |
| 6–10 | **1.012** | **0.320** | **2.752** | **1.470–5.153** | **0.002** |  | 0.610 | 0.364 | 1.841 | 0.903–3.754 | 0.093 |  | 0.403 | 0.602 | 1.496 | 0.460–4.866 | 0.503 |
| >10 | 0.178 | 0.420 | 1.194 | 0.525–2.718 | 0.672 |  | -0.112 | 0.459 | 0.894 | 0.364–2.197 | 0.808 |  | 0.352 | 0.690 | 1.421 | 0.367–5.498 | 0.610 |
| Total daily commuting time（ref: <1, h） | | | | | |  |  |  |  |  |  |  |  |  |  |  |  |
| 1–3 | -0.414 | 0.288 | 0.661 | 0.376–1.162 | 0.151 |  | -0.194 | 0.317 | 0.823 | 0.443–1.532 | 0.540 |  | 0.203 | 0.524 | 1.225 | 0.439–3.420 | 0.698 |
| >3 | -0.720 | 0.754 | 0.487 | 0.111–2.131 | 0.339 |  | 0.418 | 0.785 | 1.520 | 0.326–7.077 | 0.594 |  | **3.020** | **1.010** | **20.496** | **2.833–148.266** | **0.003** |
| Standing or walking hours per shift（ref: <4, h） | | | | | |  |  |  |  |  |  |  |  |  |  |  |  |
| 4–8 | -0.033 | 0.323 | 0.968 | 0.514–1.822 | 0.919 |  | -0.147 | 0.364 | 0.864 | 0.423–1.762 | 0.687 |  | -0.440 | 0.582 | 0.644 | 0.206–2.015 | 0.450 |
| >8 | -0.432 | 0.524 | 0.649 | 0.232–1.814 | 0.410 |  | -0.394 | 0.562 | 0.675 | 0.224–2.029 | 0.484 |  | 0.500 | 0.766 | 1.648 | 0.367–7.395 | 0.514 |
| Learning or training sessions per month（ref: None） | | | | | |  |  |  |  |  |  |  |  |  |  |  |  |
| 1–2 | **-1.202** | **0.584** | **0.301** | **0.096–0.945** | **0.040** |  | **-1.895** | **0.623** | **0.150** | **0.044–0.510** | **0.002** |  | **-2.214** | **0.836** | **0.109** | **0.021–0.563** | **0.008** |
| 3–4 | -0.395 | 0.629 | 0.674 | 0.196–2.312 | 0.530 |  | -1.090 | 0.676 | 0.336 | 0.089–1.265 | 0.107 |  | **-2.345** | **0.967** | **0.096** | **0.014–0.638** | **0.015** |
| ≥5 | **-2.963** | **0.878** | **0.052** | **0.009–0.289** | **0.001** |  | **-3.030** | **0.889** | **0.048** | **0.008–0.276** | **0.001** |  | -1.395 | 1.145 | 0.248 | 0.026–2.340 | 0.223 |
| Self-reported workload（ref: Light） | | | | | |  |  |  |  |  |  |  |  |  |  |  |  |
| Moderate | **1.296** | **0.307** | **3.654** | **2.003–6.667** | **<0.001** |  | **1.438** | **0.366** | **4.212** | **2.057–8.626** | **<0.001** |  | **1.363** | **0.695** | **3.909** | **1.002–15.256** | **0.050** |
| Heavy | 2.863 | 1.509 | 17.507 | 0.909–337.182 | 0.058 |  | **4.512** | **1.536** | **91.076** | **4.485–1849.426** | **0.003** |  | **6.670** | **1.674** | **788.152** | **29.623–20969.609** | **<0.001** |
| Intention to stay（ref: High） | | | | | |  |  |  |  |  |  |  |  |  |  |  |  |
| With concerns | 0.527 | 0.293 | 1.693 | 0.953–3.008 | 0.072 |  | **1.460** | **0.323** | **4.306** | **2.288–8.103** | **<0.001** |  | **1.258** | **0.535** | **3.519** | **1.234–10.039** | **0.019** |
| Low | 1.334 | 1.435 | 3.796 | 0.228–63.236 | 0.353 |  | 2.808 | 1.471 | 16.572 | 0.927–296.196 | 0.056 |  | **3.809** | **1.630** | **45.096** | **1.848–1100.604** | **0.019** |
| Physical workplace comfort perception（ref: Comfortable） | | | | | |  |  |  |  |  |  |  |  |  |  |  |  |
| Temperature | 0.212 | 0.501 | 1.236 | 0.463–3.298 | 0.672 |  | 0.220 | 0.515 | 1.246 | 0.454–3.420 | 0.669 |  | -0.445 | 0.703 | 0.641 | 0.162–2.540 | 0.527 |
| Humidity | -0.256 | 0.477 | 0.774 | 0.304–1.973 | 0.591 |  | -0.123 | 0.499 | 0.884 | 0.332–2.350 | 0.805 |  | -0.526 | 0.715 | 0.591 | 0.146–2.400 | 0.462 |
| Air quality | -0.429 | 0.382 | 0.651 | 0.308–1.376 | 0.261 |  | 0.141 | 0.432 | 1.152 | 0.494–2.686 | 0.743 |  | -0.328 | 0.727 | 0.720 | 0.173–2.992 | 0.651 |
| Lighting intensity | -0.138 | 0.393 | 0.871 | 0.403–1.882 | 0.725 |  | -0.634 | 0.415 | 0.531 | 0.235–1.197 | 0.127 |  | -0.714 | 0.629 | 0.490 | 0.143–1.681 | 0.256 |
| Sound intensity | **-0.734** | **0.350** | **0.480** | **0.242–0.954** | **0.036** |  | **-1.673** | **0.402** | **0.188** | **0.085–0.413** | **<0.001** |  | **-1.960** | **0.809** | **0.141** | **0.029–0.687** | **0.015** |
| Overall work environment | 0.109 | 0.472 | 1.115 | 0.442–2.814 | 0.818 |  | -0.267 | 0.500 | 0.765 | 0.287–2.039 | 0.593 |  | 0.170 | 0.774 | 1.186 | 0.260–5.406 | 0.826 |
